# Polymorphisms in O-methyltransferase genes are associated with stover cell wall digestibility in European maize (*Zea mays *L.)

**DOI:** 10.1186/1471-2229-10-27

**Published:** 2010-02-12

**Authors:** Everton A Brenner, Imad Zein, Yongsheng Chen, Jeppe R Andersen, Gerhard Wenzel, Milena Ouzunova, Joachim Eder, Birte Darnhofer, Uschi Frei, Yves Barrière, Thomas Lübberstedt

**Affiliations:** 1 Department of Agronomy, Iowa State University, Agronomy Hall, Ames, IA 50011, USA; 2 Department of Agronomy and Plant Breeding, Technical University of Munich, Am Hochanger 2, 85354 Freising-Weihenstephan, Germany; 3 Department of Genetics and Biotechnology, University of Aarhus, Research Center, Flakkebjerg, 4200 Slagelse, Denmark; 4 KWS Saat AG, Grimsehlstr 31, 37555 Einbeck, Germany; 5 Bavarian State Research Center for Agriculture, Vöttinger Str. 38, 85354 Freising-Weihenstephan, Germany; 6 Unite' de Ge'ne'tique et d'Ame'lioration des Plantes Fourrage'res, INRA, Route de Saintes, 86600 Lusignan, France

## Abstract

**Background:**

OMT (O-methyltransferase) genes are involved in lignin biosynthesis, which relates to stover cell wall digestibility. Reduced lignin content is an important determinant of both forage quality and ethanol conversion efficiency of maize stover.

**Results:**

Variation in genomic sequences coding for *COMT, CCoAOMT1*, and *CCoAOMT2 *was analyzed in relation to stover cell wall digestibility for a panel of 40 European forage maize inbred lines, and re-analyzed for a panel of 34 lines from a published French study. Different methodologies for association analysis were performed and compared. Across association methodologies, a total number of 25, 12, 1, 6 *COMT *polymorphic sites were significantly associated with DNDF, OMD, NDF, and WSC, respectively. Association analysis for *CCoAOMT1 *and *CCoAOMT2 *identified substantially fewer polymorphic sites (3 and 2, respectively) associated with the investigated traits. Our re-analysis on the 34 lines from a published French dataset identified 14 polymorphic sites significantly associated with cell wall digestibility, two of them were consistent with our study. Promising polymorphisms putatively causally associated with variability of cell wall digestibility were inferred from the total number of significantly associated SNPs/Indels.

**Conclusions:**

Several polymorphic sites for three O-methyltransferase loci were associated with stover cell wall digestibility. All three tested genes seem to be involved in controlling DNDF, in particular *COMT*. Thus, considerable variation among *Bm3 *wildtype alleles can be exploited for improving cell-wall digestibility. Target sites for functional markers were identified enabling development of efficient marker-based selection strategies.

## Background

Stover cell-wall digestibility has long been shown to be crucial for forage quality, and more recently this trait is getting more attention in relation to biofuel production. Research into bioethanol has grown significantly as a response to global warming and increasing prices of fossil fuels. The conversion of lignocellulosic biomass into fermentable sugars by the addition of enzymes has long been recognized as an alternative to the existing starch-based ethanol production [1-3]. Reduced lignin content improves cell wall digestibility due to increased accessibility of cellulose and hemicelluloses by enzymatic procedures, enabling better ethanol conversion efficiency [4]. Lignins are phenolic polymers resulting from three monolignols: *p*-coumaryl, coniferyl, and sinapyl alcohol. These monolignols derive *p*-hydroxyphenyl H, guaiacyl G, and syringyl S phenylpropanoid units, respectively, which polymerize by oxidation to form lignins [5]. In maize, lignins are predominantly comprised of G and S units (37.5 and 60.0%, respectively) [6], with a low, but noticeable content in H units which is nearly five times higher than in dicotyledonous plants.

Lignin is synthesized by the phenylpropanoid pathway [7]. Phenylalanine ammonia lyase (*PAL*) catalyzes the first step by removing ammonia from L-Phe to produce p-coumaric acid, followed by a series of enzymatic steps involving cinnamate 4-hydroxylase (*C4H*), 4-coumarate:CoA ligase (*4CL*), hydroxycinnamoyl-CoA transferase (*HCT*), *p*-coumarate 3-hydroxylase (*C3H*), caffeoyl-CoA *O*-methyltransferase (*CCoAOMT*), cinnamoyl-CoA reductase (*CCR*), ferulate 5-hydroxylase (*F5H*), caffeic acid *O*-methyltransferase (*COMT*), and cinnamyl alcohol dehydrogenase (*CAD*) catalyzing the biosynthesis of monolignols. Genes encoding these enzymes controlling the phenylpropanoid pathway in maize have been cloned [8-15].

Four brown midrib mutations in maize (*bm1, bm2, bm3*, and *bm4*) are associated with alterations of and reductions in lignin content in stover [16]. The *bm1 *mutation decreases cinnamyl alcohol dehydrogenase (*CAD*) activity [17], while the *bm3 *mutation results in reduced caffeic acid *O*-methyltransferase (*COMT*) activity [18]. The genetic events underlying *bm2 *and *bm4 *mutations are unknown.

Several studies reported improvement in the digestibility of corn silage in ruminant feeding of *brown midrib *mutants, especially *bm3 *materials [19-25]. However, negative effects of *brown midrib *genotypes have also been reported in relation to agricultural fitness, such as reduced grain and stover yield and stalk breakage [26-28]. Reduced grain yield, combined with other negative effects caused by the *bm3 *mutation is so significant that it might be difficult to produce superior maize *bm3 *hybrids in terms of agricultural fitness [27,26]. However, the same authors report large genetic variation among *bm3 *lines, and the genotype specificity among *bm3 *lines in relation to yield reduction [26,27,29].

Although many studies investigated phenotypic effects in relation to down regulation of lignin genes for maize and other species [30-36], intragenic variability of OMT genes involved in lignin synthesis have not been studied to the same extent. Ultimately, characterized QTN (Quantitative Trait Nucleotide) or QTINDEL (Quantitative Trait Insertion-Deletion) polymorphisms within these genes would allow development of Functional Markers (FM) [37]. Prerequisite for distinguishing the effects of different intragenic polymorphisms is low LD (linkage disequilibrium) in the population(s) analyzed. Nucleotide diversity, LD, and associations to forage quality traits have been studied for several genes involved in the phenylpropanoid pathway [38-41]. While LD decreased rapidly within few hundred bp in the *COMT *and *CCoAOMT2 *coding genes [40,41], LD persisted over a thousand bp in the *CCoAOMT1 *gene [40].

In this study, variation in genomic sequences coding for *COMT, CCoAOMT1*, and *CCoAOMT2 *was analyzed in relation to stover cell wall digestibility for the same panel of 40 European forage maize inbred lines investigated by Andersen et al. [38,42] for other "lignin genes" (Experiment 1). In addition, data published by Guillet-Claude et al. [40] in an association analysis of the three O-methyltransferase genes in relation to cell wall digestibility were re-analyzed (Experiment 2), based on a different statistical approach by using General Linear Model, and including population structure. The objectives were, (1) to identify associations between individual polymorphisms and four forage quality traits determined for maize stover in Exp. 1, (2) to evaluate the impact of sequence alignment parameters on the outcome of association studies, (3) to re-analyze data of Exp. 2 under consideration of population structure, and (4) to compare findings between both experiments for these three jointly analyzed genes.

## Methods

### Plant Materials and Phenotypic Analyses

The 40 lines within Exp. 1 represent a broad range of Central European forage maize germplasm, and were extremes within a larger collection of >300 maize inbreds with respect to stover cell-wall digestibility (unpublished data). Thirty-five lines originated from the current breeding program of KWS Saat AG and five lines were from the public domain (AS01, AS02, AS03, AS39, and AS40, identical to F7, F2, EP1, F288, and F4, respectively).

The inbred lines were evaluates in Grucking (sandy loam) in 2002, 2003, and 2004, and in Bernburg (sandy loam) in 2003 and 2004. The experiments consisted of 49 entries in a 7 × 7 lattice design with two replications. Nine entries from this design were consisting of not sequenced checks (such as knock-out mutants of brown midrib loci with known high cell wall digestibility). Therefore, only 40 lines were analyzed for cell wall properties and sequenced. The single row plots were 0.75 m apart and 3 m long with a total of 20 plants. The ears were manually removed and the stover was chopped 50 days after flowering. Approximately 1 kg of stover was collected and dried at 40°C. The stover was ground to pass through a 1 mm sieve. Quality analyses were performed with near infrared reflectance spectroscopy (NIRS) based on previous calibrations on the data of 300 inbred lines (unpublished results). Four traits were analyzed: Water Soluble Carbohydrate (WSC), Organic Matter Digestibility (OMD), Neutral Detergent Fiber (NDF) and digestible Neutral Detergent Fiber (DNDF). DNDF was estimated by DNDF = 100 - (IVDMD - (100 - NDF))/NDF based on Goering and Van Soest, 1970 [43]. Since the investigated traits were highly heritable [42,44], and "trait × location" interactions were not significant, replications and locations were averaged, so that each entry was represented by mean values of the cell wall traits. The results of phenotypic analysis were published previously [38]. The heritabilities of NDF and DNDF were 86.5% and 92.2%, respectively [42].

Thirty-four inbred lines were employed in Exp. 2, including public lines and ecotypes both from Europe and U.S., covering substantial variation in forage quality. The lines were evaluated in three different years: 2006, 2008, and 2009 (unpublished data of INRA Lusignan). Values of stover *in vitro *cell wall digestibility was estimated by DNDF = (100 × (ES - (100 - NDF)/NDF) [45], based on enzymatic solubility (ES) of Aufrere and Michalet-Doreau,[46].

#### DNA isolation, PCR amplification, and DNA sequencing

The inbred lines from Exp. 1 were grown in the greenhouse for DNA isolation. Leaves were harvested three weeks after germination, and DNA was extracted using the Maxi CTAB method [47]. Polymerase chain reaction (PCR) primers were developed based on maize mRNA sequences identified in GenBank by employing BLAST [48] for three genes: *COMT*, *CCoAOMT1 *and *CCoAOMT2*. PCR reactions contained 20 ng genomic DNA, primers (200 nM), dNTPs (200 μM), 1 M Betain and 2 units of Taq polymerase (Peqlab, Erlangen, Germany), in a total reaction volume of 50 μl. A touchdown PCR program was: denaturation at 95°C for 2 min, 15 amplification cycles: 45 sec at 95°C; 45 sec at 68°C (minus 0.5°C per cycle), 2 min at 72°C, followed by 24 amplification cycles: 45 sec at 95°C; 45 sec at 60°C, 2 min at 72°C, and a final extension step at 72°C for 10 min.

Products were separated by gel electrophoresis on 1.5% agarose gels, stained with ethidium bromide and photographed using an eagle eye apparatus (Herolab, Wiesloch, Germany). Amplicons were purified using QiaQuick spin columns (Qiagen, Valencia, USA) according to the manufacturer instructions, and directly sequenced using internal sequence specific primers and the Big Dye1.1 dye-terminator sequencing kit on an ABI 377 (PE Biosystems, Foster City, USA). Electropherograms of overlapping sequencing fragments were manually edited using the software Sequence Navigator version 1.1, from PE Biosystems.

Full alignments were built for *CCoAOMT1 *and *CCoAOMT2 *genes by using default settings of the CLUSTAL W program. Several Indels were present among the different *COMT *alleles in Exp. 1 [24]. Thus, four different alignment parameters were set in CLUSTAL W [49] to validate the consistency of polymorphic sites. The first alignment was based on default parameters, additional alignments were constructed by using different parameter settings in relation to gap penalty. The exon-intron structure of the three O-methyltransferase genes was estimated by alignments of genomic to mRNA sequences.

In Exp. 2, primer pairs for *CCoAOMT1 *and *CCoAOMT2 *were designed based on published cDNA sequences (accession numbers AJ242980 and AJ242981), respectively. For both genes, fragments of about 1.2 to1.3 kb were amplified, encompassing the 5'-UTR and the complete coding region [40]. Since the *COMT *promoter sequence was not available in databases, a walking-PCR procedure was performed to amplify the 5'-flanking region. Sequencing was performed for each PCR fragment in both directions by Isoprim (Toulouse, France) and MWG-Biotech (Ebersberg, Germany). The sequences containing singletons were checked by re-amplifying genomic DNA and partially re-sequencing the appropriate alleles. Contigs were constructed using SeqWeb (GCG Wisconsin Package). Sequences were aligned using CLUSTAL W [49].

#### Population structure and association analysis

Lines evaluated in Exp. 1 were genotyped with 101 simple sequence repeat (SSRs) markers providing an even coverage of the maize genome. Population structure was estimated from the SSR data by the *Structure *2.0 software [50,51]. The Q matrix estimating membership coefficients for each individual in each subpopulation was produced. A burn-in length of 50.000 followed by 50.000 iterations was applied. The Admixture model was applied with independent allele frequencies.

Association between polymorphisms and mean phenotypic values were performed by the General Linear Model (GLM) analyses in *TASSEL*. The Q matrix produced by *Structure *was included as covariate in the analysis to control for populations structure. The polymorphisms were determined as significant for p-adj_Marker (based on 10.000 permutations) equal to 0.05 or less. p-adj_Marker is a permutation based experiment-wise error rate which controls the error rate over all the markers tested.

The same parameters were applied to perform the Logistic Regression in *TASSEL*. In this association analysis a logistic regression ratio test is used to evaluate associations involving quantitative traits while controlling for population structure [52]. The trait values permuted relative to the fixed haplotypes were recalculated for 10.000 permutations.

Associations were also tested by the Mixed Linear Model (MLM) in *TASSEL *[53]. The MLM accounts not only for overall population structure (Q), but also finer scale relative kinship (K). Loiselle kinship coefficients [54] between lines (a K matrix) were estimated by the SPAGeDI software [55] based on the SSR data mentioned above. Negative values between two individuals in the K matrix were set to zero. The Bonferroni Step-down correction [56] was applied to correct for multiple testing as the p-values for the MLM analysis are expressed on a single marker basis in TASSEL.

Significant intron polymorphisms in Exp.1 were analyzed for alterations in motif sequences, using the inbred line W64 as reference sequence (AY323283). Differential splicing was tested by comparing expressed sequences with genomic sequences. The discrimination of favorable and unfavorable alleles at each significant associated polymorphic site at *COMT *was done by grouping lines by alleles and averaging DNDF values within the same group.

Guillet-Claude et al. [40] tested for associations between cell wall digestibility and polymorphic sites of *COMT*, *CCoAOMT1*, and *CCoAOMT2 *by performing Multiple Linear Regression without considering population structure. Our re-analysis included population structure in the GLM analysis of TASSEL. The P-value of all individual polymorphisms (including singletons) was estimated based on 10,000 permutations. Population structure data were obtained from Camus-Kulandaivelu et al. [57], where five sub-populations were found using 55 SSR loci in *Sructure *[50,51].

## Results

### Genetic diversity within *COMT*, *CCoAOMT1*, and *CCoAOMT2*

The analysis of overlapping sequences of common inbred lines between Exp.1 and Exp.2 (F2, F4, F7, F288, EP1, and W64) revealed consistency of sequences for the *COMT *gene across both experiments. Only one SNP each was different among overlapping sequences of F4 and EP1 inbred lines, respectively (Table 1). For *CCoAOMT1*, F2 and F7 sequences were identical in both experiments, whereas between two (F288) and 13 (EP1) polymorphisms were identified for the other three inbred lines (Table 1). For the CCoAMT2 gene, only two inbred lines were common between both studies. One SNP and one large Indel were identified for F288, whereas sequences perfectly matched for EP1. For each of the three genes, sequences of lines used in both experiments, were in all cases most similar across both experiments: even when comparing the EP1 *CCoAOMT1 *allele from Exp. 1 with all alleles in Exp. 2, the Exp. 2 EP1 allele was the most similar sequence to Exp. 1 EP1 among all Exp. 2 sequences.

**Table 1:** Comparison of sequences of common inbred lines in Exp. 1 and Exp 2

	*COMT*		*CCoAOMT1*		*CCoAOMT2*	
Inbred lines	Bp Overlap	Differences	Bp Overlap	Differences	Bp Overlap	Differences
F2	2051	0	1326	0	-	-
F4	1947	1 SNP	1327	5 SNP, 1 Indel	-	-
F7	2090	0	1249	0	-	-
F288	2052	0	1328	1 SNP, 1 Indel	655	1 SNP, 1 Indel
EP1	2115	1 SNP	1254	9 SNP, 4 Indel	751	0
W64	2088	0	-	-	-	-

Analysis of haplotype diversity for the 40 inbred lines employed in Exp. 1 revealed 13, 9, and 6 haplotypes for *COMT*, *CCoAOMT1*, and *CCoAOMT2*, respectively (Table 2). The range of haplotype means for all four stover quality traits was larger for *COMT *compared to the other two genes (Table 2). A total number of 26, 12, and 14 haplotypes was previously identified by Guillet-Claude et al. [40], for *COMT*, *CCoAOMT1*, and *CCoAOMT2*, respectively. When sequences from both experiments were jointly analyzed for haplotype numbers, 38 haplotypes were discriminated for *COMT*, and 15 haplotypes each for *CCoAOMT1*, and *CCoAOMT2 *(data not shown).

**Table 2:** Number of haplotypes based on single nucleotide polymorphisms (SNPs) in the *COMT*, *CCoAOMT1*, and *CCoAOMT2 *genes of maize, and minimum, maximum and variance of phenotypic values of lines representing individual haplotypes

		WSC			NDF			OMD			DNDF		
Gene	No Haplotypes	Min	Max	Range	Min	Max	Range	Min	Max	Range	Min	Max	Range
*COMT*	13	13.69	23.68	9.99	52.92	65.90	12.98	61.55	76.65	15.1	42.18	59.98	17.8
*CCoAOMT1*	9	16.04	23.28	7.24	52.93	61.93	9.00	68.02	75.55	7.53	50.20	59.98	9.78
*CCoAOMT2*	6	13.28	20.69	7.41	54.95	63.03	8.08	71.00	74.46	3.46	54.07	59.98	5.91

#### Association analysis of *COMT *in Experiment 1

Varying alignment lengths and number of polymorphic sites were observed when four different parameter sets were applied for aligning *COMT *by CLUSTAL W. By GLM based on the first alignment (default settings), 16 polymorphisms were significantly associated with DNDF, whereas alignment settings 2, 3, and 4 identified 13, 14, and 14 polymorphic sites, respectively (Table 3), significantly associated with DNDF. Different gap opening and gap extension penalties lead to different Indel numbers and sizes. Some polymorphisms were observed to change positions, others were created or vanished. Only eight polymorphic sites showing significant associations with DNDF were in common among all four alignments.

All subsequent analyses were performed for default sequence alignment settings, in order to be able to compare findings reported here with previous studies [38,42]. In Exp. 1, a total number of 16 *COMT *polymorphic sites were significantly associated with DNDF using GLM analysis: 9 SNPs and 7 Indels (Table 4). The same sites were identified by MLM analysis, with the exception of one SNP located at 1439 bp and one Indel located at 1638 bp based on the reference sequence AY323283 of Genbank. Logistic regression analysis in TASSEL identified a larger number of significant polymorphic sites in association with DNDF: 16 SNPs and 9 Indels, respectively (Table 4).

**Table 3:** Significantly associated polymorphic sites of *COMT *with DNDF, identified by GLM, based on alignments resulting from four different parameters settings of CLUSTAL W

Site	Alignments			
	1 (2461 bp)	2 (2517 bp)	3 (2508 bp)	4 (2553 bp)
**701**	X	X	X	X
**737**	X	X	X	X
**781**			X	
**782**	X	X		X
**787**	X		X	X
**793**				X
**824**		X	X	X
**845**	X			X
**855**	X			X
**897**		X		
**962**	X	X	X	X
**1010**	X	X	X	X
**1059**	X	X	X	X
**1240**	X	X	X	X
**1351**	X			
**1352**	X			
**1354**	X			
**1356**		X		
**1388**	X			
**1417**			X	
**1419**			X	
**1432**			X	
**1562**		X		
**1612**	X	X	X	X
**1619**	X	X	X	X

Site numbers denote bp alignment positions of individual SNPs and starting positions of Indels based on reference sequence AY323283 of Genbank.

**Table 4:** Polymorphic sites of *COMT *associated with DNDF identified by GLM, MLM, and Logistic Regression tests for DNDF

Site	Snp/Indel	E/I	aa change	GLM	MLM	REG	Other trait*
1233	C-G	I	-	-	-	-	WSC
1235	A-T	I	-	-	-	-	WSC
1236	C-G	I	-	-	-	-	WSC
1240	C-T	I	-	-	-	-	WSC
1243	10	I	-	-	-	-	WSC
1261	A-G	I	-	-	-	-	WSC
1296	C-T	I	-	X	X	X	OMD
1331	C-T	I	-	X	X	X	OMD
1377	A-T	I	-	X	X	X	OMD
1381	7	I	-	X	X	X	OMD
1439	A-C	I	-	X	-	X	-
1449	4-8	I	-	X	X	-	-
1547	6	I	-	X	X	X	OMD
1589	A-G	I	-	X	X	X	OMD
1638	1	I	-	X	-	X	OMD
1811	6	I	-	X	X	X	OMD
1902	1	I	-	-	-	X	-
1907	3	I	-	-	-	X	-
1916	A-G	I	-	-	-	X	-
1917	A-C	I	-	X	X	X	-
1918	A-G	I	-	X	X	X	-
1919	C-T	I	-	-	-	X	-
1920	28	I	-	X	X	X	OMD
1948	C-T	I	-	-	-	X	-
1952	C-T	I	-	-	-	X	-
1953	A-T	I	-	-	-	X	-
1954	77	I	-	X	X	X	OMD
2032	A-C	I	--	-	-	X	-
2103	C-T	E2	Ser - Pro	-	-	X	OMD
2178	C-G	E2	His - Asp.	X	X	X	OMD
2185	C-G	E2	Arg - Pro	X	X	X	-
2693	A-C	E2	Syn.	-	-	-	NDF

*All polymorphic sites associated with WSC, OMD and NDF were identified only by Logistic Regression. E: exon; I: intron; aa: Amino Acid; Syn.: Synonymous substitution

Site numbers denote bp alignment positions of individual SNPs and starting position of Indels based on reference sequence AY323283 of Genbank.

**Figure 1:**
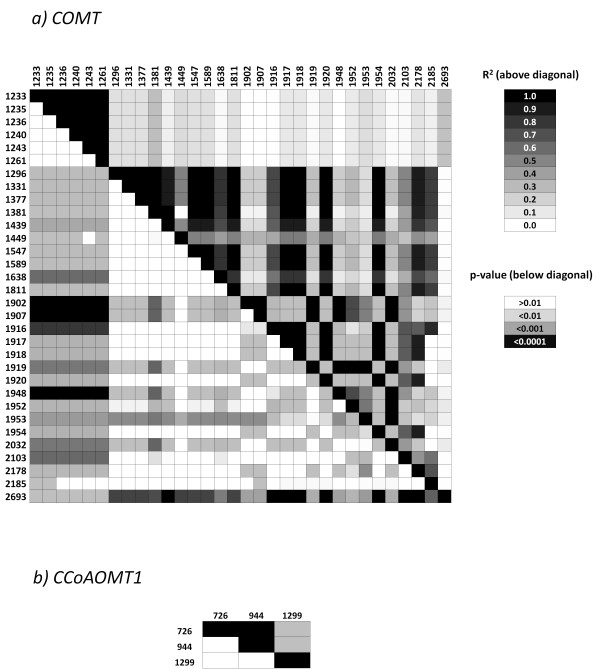
**Linkage disequilibrium among significantly associated SNPs and Indels estimated by TASSEL, for DNDF, OMD, NDF and WSC of a) *COMT*, b) *CCoAOMT1 *in Experiment 1**.

No polymorphic sites were observed to be significantly associated with DNDF in the first exon, and most of the polymorphic sites identified for this trait were located in the intron, where 13 SNPs and 9 Indels were detected. In the second exon, three SNPs were significantly associated with DNDF in positions 2103, 2178, and 2185 bp, each leading to amino acid substitutions (Ser/Pro, His/Asp, and Arg/Pro, respectively). Most of the significantly associated polymorphic sites identified for DNDF were in high LD (Fig. 1).

No significant associated SNP or Indel was identified between *COMT *and NDF, OMD, and WSC by MLM or GLM (Table 4). All significantly associated polymorphic sites for these traits were identified by Logistic Regression. For NDF, a synonymous SNP was identified in the second exon of the *COMT *gene. Low values of LD were observed between this SNP and other polymorphic sites associated with DNDF, OMD, and WSC (Fig. 1). All polymorphic sites (six SNPs and six Indels) associated with OMD matched positions of DNDF associated polymorphic sites, most of them located in the intron. Only two SNPs (2103 and 2178 bp) were identified in the second exon of the gene, both causing changes in amino acid sequences (Ser/Pro and His/Asp, respectively). All polymorphic sites identified for WSC were located in the intron: five SNPs and one Indel with high LD among each other (Fig. 1). Six singletons in complete LD located at positions 1064, 1070, 1071, 1072, 1073, and 1074 bp were identified by GLM to be significantly associated with all four traits, but not included in Table 4.

**Figure 2:**
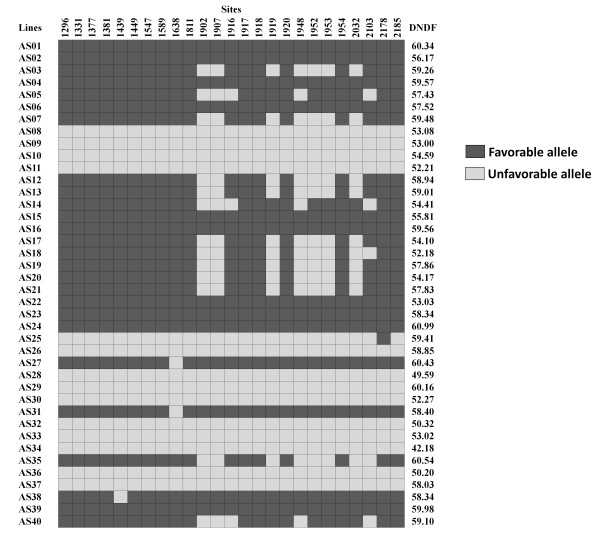
**Discrimination of favorable and unfavorable *COMT *alleles in relation to average *DNDF *for lines in Exp.1**.

Eight significantly associated Indels in the *COMT *intron affected five motifs representing binding sites for transcription factors RAV1, GAmyb, and DOFs 1, 2, and 3. Tests for differential splicing did not reveal divergent patterns of exon inclusion (data not shown). Across polymorphisms significantly associated with DNDF, lines containing mostly favorable alleles had on average higher DNDF values than lines containing mostly unfavorable alleles (Fig. 2). However, one line (AS22) with all favorable alleles had a low DNDF value and three lines (AS26, AS29 and AS37) containing only unfavorable alleles had high DNDF values.

#### Association analysis of *CCoAOMT1 *and *CCoAOMT2 *in Experiment 1

Association analysis for *CCoAOMT1 *revealed substantially fewer polymorphic sites associated with the traits investigated as compared to *COMT*. Two SNPs were identified by GLM: one SNP was associated with NDF and located in the third intron, and a second SNP was associated with both NDF and OMD, located in the fourth intron. One additional SNP identified by Logistic Regression (Table 5) associated with WSC was identified in the fifth exon, leading to amino acid substitution. High LD was observed between SNPs of sites 726 and 944, while low LD was observed between site 1299 and sites 726 and 944 bp (Fig. 1).

**Table 5:** Significantly associated polymorphic sites of *CCoAOMT1 *and *CCoAOMT2 *genes identified by GLM, MLM and Logistic Regression (REG) with NDF, OMD, and WSC, respectively

Gene	Trait	Site	Snp/Indel	E/I	aa change	GLM	MLM	REG
*CCoAOMT1*	NDF	726	C-T	I3	-	X	-	-
	NDF	944	C-T	I4	-	X	-	-
	OMD	944	C-T	I4	-	X	-	-
	WSC	1299	C-G	E5	Ala - Gly	-	-	X

*CCoAOMT2*	WSC	404	1	I2	-	-	-	X
	WSC	414	4	I2	-	X	-	X

E: exon; I: intron; aa: Amino Acid.

Site numbers denote bp alignment positions of individual SNPs and starting position of Indels. (Based on reference sequences AY323264 and AY279022, for *CCoAOMT1 *and *CCoAOMT2*, respectively).

Only two Indels showed significant trait associations for *CCoAOMT2 *by Logistic Regression, and only one was also detected by GLM (Table 5). Both Indels were located in the second intron of the *CCoAOMT2 *gene, and both were associated with WSC. Both polymorphisms were in complete LD. intron polymorphisms of *CCoAOMT1 *and *CCoAOMT2 *did not cause changes in known motifs, and were not found to cause differential splicing.

#### Association analysis of *COMT*, *CCoAOMT1*, and *CCoAOMT2 *in Experiment 2

Guillet-Claude et al. [40] identified polymorphic sites of *COMT *associated with DINAGZ, all located in non-coding regions. No association was found between polymorphisms within *CCoOAMT1 *and DINAGZ, while an 18-bp Indel associated with DINAGZ was identified for *CCoAOMT2*. DINAGZ and DNDF are both estimates of CWD based on *in vitro *enzymatic solubility, differing on how the fully digestible part of the non-cell-wall part is computed. The correlation between the two traits is high (nearly 0.90, INRA Lusignan unpublished data). For this reason, the association results from Guillet-Claude et al. [40] should be phenotypically comparable to our analysis (using DNDF).

After taking population structure into account, five sub-populations were identified for this line panel by Camus-Kulandaivelu et al. [57]. In total 14 polymorphic sites showed significant associations with DNDF for the three investigated genes (Table 6). Thirteen of those polymorphic sites were singletons, and most of them were located in non-coding regions.

**Table 6:** Significant polymorphic sites identified in Exp. 2 in comparison to Exp. 1

Gene	Exp.2 polymorphisms				Exp.1
		
	Site	Region	SNP/Indel	aa Change	p-value
	342	Prom	A-T	-	-
*COMT*	659*	Prom	1	-	-
	749*	E1	6	-	0.99
	1948*	I1	G-T	-	0.03
	1981*	I1	C-T	-	0.92

*CCoAOMT1*	704*	I3	3	-	-
	734*	I3	2	-	1
	944*	I4	C-G	-	0.01
	956*	I4	11	-	-
	972*	I4	A-G	-	-

*CCoAOMT2*	187*	E1	A-G	His - Arg	-
	688*	I3	A-G	-	-
	717*	I3	C-T	-	-
	720*	I3	C-T	-	-

E: exon; I: intron; Prom: promoter; aa: Amino Acid; Syn.: Synonymous substitution; *: singleton

Site numbers denote bp alignment position of individual SNPs and starting position of Indels based on the reference sequences AY323283, AY323264 and AY279022, for *COMT*, *CCoAOMT1 *and *CCoAOMT2*, respectively.

For *COMT*, three SNPs and two Indels were identified by GLM. The SNP located at position 1948 bp was also identified as significantly associated with DNDF in Exp. 1. This SNP was observed to be in high LD with another polymorphic site at position 1981 bp, not identified in Exp.1, and low LD with the other three trait-associated sites identified in Exp. 2. The two polymorphic sites at positions 749 bp and 1981 bp were also present in Exp.1, but not significantly associated with any of the four traits analyzed. Two polymorphisms located in the promoter (at positions 342 and 659 bp) were not polymorphic in Exp.1.

Contrary to the findings of Guillet-Claude et al. [40], two SNPs and three Indels showed significant associations between *CCoAOMT1 *and DNDF. One SNP located at position 944 bp was also found to be significantly associated with NDF and OMD in Exp. 1. This SNP was in high LD with Indels at positions 704 and 734 bp, and in low LD with polymorphic sites at positions 956 and 972 bp. The Indel at positon 734 bp was also observed in Exp.1, but it was not significantly associated with any four traits investigated in Exp. 1. Four SNPs in *CCoAOMT2 *sequence were significantly associated with DNDF. All polymorphisms were singletons not identified in Exp.1, and three of them were located in non-coding regions.

## Discussion

### Impact of sequence alignments and association analysis methods on results of association studies

Changing alignments parameters for gap opening and gap extension costs leads to the creation of alignments with different Indel sizes and frequencies [58]. Different studies investigated consequences of different alignments parameters settings in the outcome of phylogenetic studies [59-62]. However, little is known about the effect of different alignment settings in association analysis. Our results demonstrate that different sets of polymorphisms are identified by association analyses, depending on alignments settings (Table 3). This is especially true for sequences with multiple Indels as observed for *COMT*, where choosing optimal alignment parameters might be difficult. Comparison of different alignments varying for gap costs may be used to identify the most consistent significantly associated polymorphic sites.

In this study, cell wall traits of two relatively small populations (40 and 34 lines) were tested for associations with two large sets of polymorphic sites (SNPs and Indels). The large number of independent variables (SNPs/Indels) in relation to number of dependent variables (lines/phenotypic data) increases the chances of false positive associations due to overfitting [63]. However, we believe that high heritability values of the cell wall traits analyzed [42,44] and stringent significance levels estimated by 10.000 permutations limit the rate of false positives [64].

The control of systematic differences due to population structure and kinship has also been suggested to avoid spurious associations and for greatest statistical power in association studies [53,65]. In this study, two of our models (GLM and Logistic Regression) were controlling for population structure, and the mixed model (MLM) approach was controlling for both population structure and kinship. Most of the lines evaluated in this study could be clearly identified as Flint or Dent lines (see Figure five: Andersen et al. [38]).

Across the investigated genes in this study, most of the significantly associated polymorphisms were detected by Logistic Regression, followed by GLM and MLM. A few previous studies compared results of association analyses from these methods. Andersen et al. [66] in an attempt to validate associations of the *Dwarf8 *locus identified by Thornsberry et al. [52], identified a larger number of polymorphisms associated with flowering time when using GLM (without control of population structure) as compared to Logistic Regression (with control of population structure). According to the authors, GLM identified more significant polymorphisms, because variation of causal alleles was closely correlated with population structure. In corroboration with our results, Andersen et al [42] observed a larger number of significant polymorphisms by GLM analysis accounting for population structure as compared to MLM accounting for both population structure and kinship corrections. Fewer significant polymorphisms identified by MLM are expected, as MLM also corrects for kinship, and significant polymorphisms identified in GLM but not in MLM might thus reflect identity of polymorphisms by descent. Investigating three QTL in 277 diverse maize inbred lines, Yu et al. [53] concluded that the model accounting for both population structure (Q) and relative kinship (K) gave the best approximation to the cumulative distribution of p-values when compared to models containing only K, only Q and a simple model (in which family structure is ignored).

Across the three genes and four traits within Exp. 1, 13 polymorphisms were consistent across the three methods, while one polymorphism was identified by both GLM and MLM, 3 by both GLM and Logistic Regression. No significant polymorphic sites were consistent between both MLM and Logistic Regression. Logistic Regression was the method detecting the highest number of significant polymorphisms in our study. With this method, permutation tests are performed for individual markers, not controlling for experiment-wise error rates. Consequently, a larger number of false positives are expected for this method of analysis when compared to analyses that control for experiment-wise error, like GLM in our study. Logistic Regression (and GLM in one occasion) revealed significant associations between the three OMT genes with WSC, with none of these three genes being involved in biosynthesis of soluble carbohydrates (Tables 4 and 5). However, we were able to identify one SNP in Exp.1 by Logistic Regression (but not by the other two methods) that was consistent with Exp. 2 (SNP at position 1948 of the *COMT *gene, Table 6). Moreover, many polymorphisms identified by Logistic Regression were also identified by GLM and MLM when considering p-values between 0.05 and 0.10, especially for associations between *COMT *with NDF, OMD, and WSC (data not shown). It can be inferred that these QTN/Indels are at the threshold of being detectable, and even small changes in statistical methods or parameters might lead to (non-) significance of these sites. Therefore, significant polymorphisms identified by association analyses, need to be validated by independent experiments (ongoing).

### Consistency of association results across experiments

Two SNPs were identified as significantly associated in both datasets (Exp.1 and Exp.2). The SNP at position 1948 bp in the *COMT *intron was significantly associated with DNDF. At this C/T polymorphic site, a sequence motif for a DOF3 zinc finger transcription factor was identified for the allele with the T base present. The second SNP consistent across experiments was located in the site 944 bp at the fourth intron of *CCoAOMT1*. This SNP did not cause motif or splicing alterations, but it was significantly associated with both NDF and OMD in Exp.1. The consistency of these two SNPs across experiments makes them excellent candidates for development of FMs.

In addition, three polymorphic sites identified in Exp. 2 were also observed in Exp.1, but not significantly associated with the investigated traits. The relative low number of common polymorphic sites between Exp.1 and Exp.2 can in part be explained by the different regions of genes that each experiment investigated. The overlapping region of each gene common to both studies was not representing the whole gene. Moreover, for some lines overlapping sequences were not completely identical in both studies (Table 1), most likely due to residual heterozygosity as the "same" lines in both studies had in all cases the most similar sequences. In addition, most of the significantly associated polymorphic sites identified in Exp. 2 were singletons, which have a low probability of being indentified across datasets. Finally, Exp. 1 and 2 were conducted in quite different environments in Germany and France, respectively, which might explain for the different performance of the "same lines" with regard to CWD in both experiments.

### Genes controlling forage quality

Most of the significant SNPs/Indels identified in Exp. 1 were identified for *COMT *(44 significant polymorphisms) while a smaller number of sites was identified for *CCoAOMT1 *and *CCoAOMT2 *(three and two significant polymorphic sites, respectively). Guillet-Claude et al [40] also observed a larger number of polymorphisms for *COMT *compared to *CCoAOMT1 *and *CCoAOMT2*. In both experiments, the alignment sequence size of *COMT *was approximately two times larger than *CCoAOMT1 *and *CCoAOMT2*, whereas the number of significantly associated polymorphisms for *COMT *were substantially higher than 2-fold.

Andersen et al. [42] investigated the same set of 40 lines for six other "lignin genes" in relation to NDF, IVDOM, and DNDF, and identified significant associations for *4CL1*, *C3H*, and *F5H *genes. The DNDF range between haplotypes within these three genes was largest for C3H (10.0%), followed by F5H (7.7%) and 4CL1 (2.3%). *COMT *haplotypes differed for DNDF by 17.8% (Table 2). Larger differences between haplotypes suggest that *COMT *is a promising candidate gene to derive markers affecting stover cell-wall digestibility. Moreover, down regulation of *COMT *in Arabidopsis, poplar, alfalfa and maize affecting the lignin content/structure support the strong effect of this gene on variation for cell wall digestibility [30-36].

### Polymorphisms associated with cell wall digestibility

**Figure 3:**
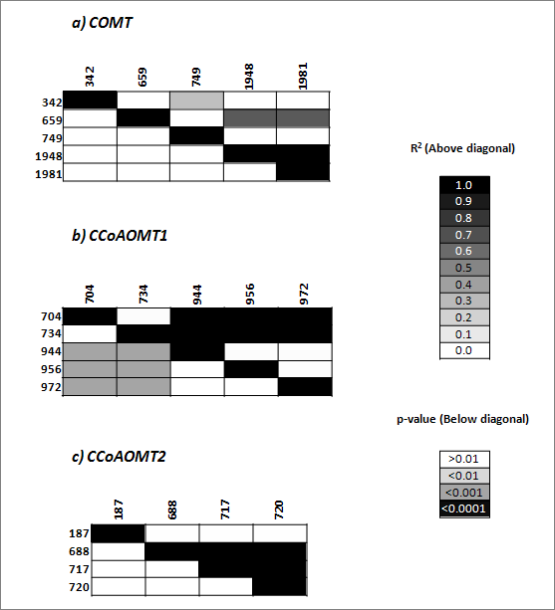
**Linkage disequilibrium among significantly associated SNPs and Indels estimated by TASSEL, for DNDF of a) *COMT*, b) *CCoAOMT1 *c) *CCoAOMT2 *in Experiment 2**.

The association analyses revealed a considerable number of SNPs and Indels associated with cell wall traits for the genes analyzed. Across experiments, methodologies and genes, 11 significant QTNs/Indels were located in coding regions and 52 significant polymorphisms were located in non-coding regions. Some of the significant associations identified in this study may reflect a statistical artifact. The probability of false-positive associations is higher in case of rare alleles and cases where a polymorphic site was only detected with one of the alignments. In addition, several of the identified significant associations can likely be explained by high LD to closely linked causal QTN/QTINDELs. Within an unresolved linkage block, likely only one or few polymorphisms are causative (Figs. 1 and 3). Polymorphisms causing amino acid changes are promising candidates for causative QTN/QTINDELs. The SNPs identified to cause amino acid changes in positions 2178 and 2185 bp of *COMT *(Table 4) were identified by all statistical methods, being, therefore, strong candidate sites to cause DNDF variation.

Several significantly associated polymorphisms located in non-coding regions have been reported. Andersen et al. [42] identified two respective SNPs in the F5H gene as significantly associated with NDF and DNDF. Wilson et al. [67] identified significant intron polymorphisms associated with kernel composition traits for *sh1 *and *sh2 *genes. Polymorphisms located in introns can be "causative" if affecting transcript abundance (expression and stability) of genes [68]. The analysis of motif alterations revealed eight significant associated Indels in *COMT *introns disrupting the sequence of five motifs, which represent binding sites for transcription factors RAV1, GAmyb, and DOFs 1, 2, and 3. DOF-type transcription factors are well known for their function as transcriptional activators or repressors of tissue-specific gene expression. More recently, they have been identified as putative regulators of lignin biosynthetic genes, based on the microarray analyses of Arabidopsis ectopic lignification mutants [69]. Therefore, although polymorphisms in positions 1439, 1920, 1948, 1952, 1953, and 1954 bp are located in the *COMT *intron, they might play an important role as cis-regulators of gene expression, causing changes in the lignin content and variation in DNDF values. intron polymorphisms might also be part of splicing signals that could cause differential splicing encoding structurally and functionally distinct protein products. However, no divergent patterns of exon inclusion were observed for the three genes analyzed (data not shown).

## Conclusions

Lines containing favorable alleles at putative QTN or QTINDELs were generally, but not always associated with high phenotypic values for cell wall digestibility. As cell wall digestibility is a polygenic inherited quantitative trait, likely unfavorable alleles at other loci affecting this trait are responsible for this finding, either by strong main effects or interaction with *COMT*. In agreement with these observations, Barriere et al. [70] identified conventional inbreds with equally high cell wall digestibility as *bm3 *lines, supporting that other genes can have strong effects on this trait. Identifying and combining favorable alleles from multiple genes affecting cell wall digestibility is, therefore, mandatory for functional marker-based improvement of stover for forage and biofuel usage.

## List of abbreviations

CCoAOMT: caffeoyl-CoA *O*methyltransferase; *COMT*: caffeic acid *O*-methyltransferase; DNDF: digestibility of neutral detergent fiber; Indel: insertion-deletion polymorphism; LD: linkage disequilibrium; NDF: neutral detergent fiber; OMD: Organic Matter Digestibility; SNP: single-nucleotide polymorphism; WSC: water soluble carbohydrates;

## Authors' contributions

EAB prepared the manuscript. EAB, JRA, and YC performed data analysis. IZ carried out allele sequencing. GW contributed to experimental design. BD and JE provided phenotypic data. MO provided the SSR data and together with GW contributed to experimental design. UKF performed sequence alignments. YB provided data from Exp.2 and reviewed the manuscript. TL coordinated the project and together with EAB prepared the manuscript. All authors read and approved the final manuscript.
